# Allergic diseases of the skin and drug allergies – 2031. Effectiveness of autologous serum therapy in patients of chronic urticaria: a randomized, single-blind, controlled trial

**DOI:** 10.1186/1939-4551-6-S1-P117

**Published:** 2013-04-23

**Authors:** Panchami Debbarman

**Affiliations:** 1 Department of Dermatology, Medical College, Kolkata, Kolkata, India

## Background

Chronic urticaria (CU) is a vexing problem to clinicians and patients alike. Search for newer modalities which can reduce pill burden is a felt need. The scope of Autologous serum therapy (AST) thus needs evaluation.

## Methods

Patient blind, parallel group, randomised, controlled study of two study arm. 54 patients were given AST (GroupA) and control group of 57 patients given injection normal saline (placebo) (GroupB). AST/Placebo was given every week for 9 weeks and then followed-up for another 3 weeks. Levocetrizine was advised to be taken SOS in both groups. AU was diagnosed by autologous serum skin test. Urticaria total severity score (TSS) and Bengali version of Dermatologic life quality index (DLQI) was used as primary efficacy variable. Mann-Whitney U test and Freidman’s ANOVA followed by post hoc Dunn’s test was used for analysis.

## Results

TSS was comparable (p>0.05) at baseline in Group A (17.74±0.44) and Group B (17.31±0.66). TSS showed significant improvement (p<0.001) from baseline 7^th^ week (12.56±2.24) onwards in group A and 8^th^ week (13.29±1.80) onwards in group B which continued till end-of-treatment visit. Between group comparison showed significant improvement (p<0.001) 4^th^ week onwards which persisted till 12^th^ week. DLQI showed significant improvement (p=0.006) in Group A than Group B at the end of study. Both AU and non-AU patients showed comparable (p>0.05) improvement of TSS.

## Conclusions

AST shows promise in treatment of urticaria regardless of the autoimmune nature.

